# Global, Regional, and National Prevalence for Type 2 Diabetes Among Women of Childbearing Age, 1992–2021: An Age–Period–Cohort Analysis Based on the Global Burden of Disease Study 2021

**DOI:** 10.1155/jdr/2197672

**Published:** 2026-01-29

**Authors:** Zhongyan Xu, Mengting Liu, Miaoran Chen, Xinying Shen, Xinying Hao, Quan Yun, Yunzhou Zheng, Yan Cui, Jun Qiao, Fukun Wang

**Affiliations:** ^1^ Clinical Laboratory, Bethune International Peace Hospital, Shijiazhuang, Hebei, China; ^2^ Department of Breast Surgery, The Second Hospital of Shanxi Medical University, Taiyuan, Shanxi, China, sxmu.edu.cn; ^3^ Department of Nephrology, Shanxi Kidney Disease Institute, Second Hospital of Shanxi Medical University, Taiyuan, Shanxi, China, sxmu.edu.cn; ^4^ Department of Pharmacology, School of Medicine, Southern University of Science and Technology, Shenzhen, Guangdong, China, sustc.edu.cn

**Keywords:** age–period–cohort analysis, Global Burden of Disease Study 2021, prevalence, Type 2 diabetes mellitus, women of childbearing age

## Abstract

In 2021, global T2DM prevalence among WCBAs reached 73.86 million (95% UI: 63.43–85.32 million), with China, India, and the United States leading. Greenland exhibited the largest rise in age‐standardized prevalence (7.93%), with an annual increase of 11.32% (95% UI: 8.33%–14.39%). From 1992 to 2021, prevalence rose in 195 countries, declined in eight, and remained stable in one. Prevalence increased with age, peaking in women aged 45–49, and was higher in low‐SDI regions, which also experienced sharper increases. Period and cohort effects worsened globally, particularly in low‐SDI regions. Population growth drove burden increases in low‐SDI areas, while epidemiological factors dominated in high‐SDI regions. T2DM‐related visual impairment was more severe in low‐ and medium‐SDI regions. Projections for 2022–2030 predict unfavorable increasing trends. T2DM prevalence among WCBAs has steadily risen since 1992, with worsening inequalities and healthcare disparities. Projections to 2030 underscore the need for targeted prevention and treatment strategies, particularly in low‐ and medium‐SDI regions.

**Summary**

• *What is already known on this topic*: Type 2 diabetes mellitus (T2DM) among women of childbearing age (WCBAs) presents severe reproductive health risks. Despite existing studies on T2DM prevalence among WCBAs in specific regions, a comprehensive global analysis of the T2DM burden along with age, period, and birth cohort effects is still lacking.
• *What this study adds*: Despite the development of medical care and technology, T2DM prevalence among WCBAs significantly increased globally in the past three decades and was predicted to continue rising before 2030. The 45–49‐year group was most susceptible. And the absolute health inequality has worsened, with the T2DM prevalence gap between high‐ and low‐SDI countries widening substantially over the study period. During medical practice, visual impairments need more attention.
• *How this study might affect research, practice, or policy*: This study provides a comprehensive perspective on the temporal trends considering age–period–cohort effects, global inequality, and future prediction of T2DM prevalence among WCBAs. Corresponding policy development to guide medical resource allocations to reduce the disease burden is urgently needed. In low‐SDI countries, population growth is the primary driver of the increasing T2DM burden. Therefore, policy initiatives should prioritize integrating T2DM screening into existing family planning and maternal health services. This would allow for early detection and management, helping to reduce the long‐term health consequences associated with T2DM. In contrast, high‐SDI countries face a different set of challenges driven by epidemiological changes, including rising obesity rates and sedentary lifestyles. In these regions, policy efforts should focus on providing prepregnancy counseling for obese women, regulating unhealthy foods more strictly, and promoting physical activity among adolescent girls to curb the early onset of obesity and T2DM.

## 1. Introduction

Diabetes is the seventh most prevalent noncommunicable disease worldwide [1], with T2DM comprising about 90% of cases [2]. Women, particularly those of childbearing age, are more susceptible to T2DM than men due to socioeconomic disparities, patterns of adipose tissue distribution, and hormonal fluctuations, especially estrogen, during menopause [3]. The United Nations′ Sustainable Development Goal to lower the global maternal mortality ratio to under 70 per 100,000 live births by 2030 aligns with the World Health Organization′s focus on improving maternal health outcomes [4]. However, T2DM among WCBAs presents severe reproductive health risks, including delayed puberty and menarche, irregular menstrual cycles, decreased fertility, adverse pregnancy outcomes, and potentially premature menopause [5]. These complications can impair female fertility and elevate maternal morbidity and mortality. T2DM not only poses serious health risks to WCBAs, but also its disease burden in WCBAs cannot be ignored. A systematic review and meta‐analysis in the Middle East and North Africa found a 7.5% prevalence of T2DM (95% confidence interval [CI]: 6.1%–9.0%) among WCBAs [6]. Despite the significant implications of these findings, a global analysis of T2DM prevalence among WCBAs is still lacking.

An in‐depth investigation into the age, period, and birth cohort effects is essential to better understand T2DM prevalence among WCBAs. Each of these factors offers unique insights into the risk profile of T2DM. First, considering age, T2DM risk varies significantly across women′s life stages [7]. For example, pregnancy elevates the risk of T2DM due to metabolic stress, and menopause introduces factors such as upper‐body fat accumulation and insulin resistance, which further increase susceptibility [8]. Second, T2DM prevalence trends are influenced by period effects, as socioeconomic development and health policies over time impact factors like neonatal birth weight, a recognized predictor of T2DM risk in later life [9]. Finally, birth cohort effects may also shape T2DM trends in WCBA populations. Shifting external conditions, lifestyle changes, and unique generational experiences contribute to variations in T2DM prevalence across cohorts [10]. While these associations offer a basis for understanding T2DM risk in WCBAs, further analysis is needed to accurately quantify the combined impact of age, period, and cohort effects on the disease′s prevalence in these populations.

Our research addresses a key gap in global burden reports and makes three significant contributions [11]. First, we conducted a longitudinal assessment focusing specifically on WCBAs (15–49 years), a group that is particularly vulnerable to the reproductive and intergenerational health effects of T2DM. Second, we moved beyond simply describing trends by applying the age–period–cohort (APC) model, which allows us to identify potential etiological drivers of T2DM prevalence and clarify underlying patterns. Finally, we integrated an inequality assessment and forecasting through 2030 to examine the different factors driving the disease burden across sociodemographic index (SDI) environments.

Despite existing studies on T2DM prevalence among WCBAs in specific regions, a comprehensive global analysis of the T2DM burden still needs to be developed. Moreover, it is essential to conduct cross‐national comparisons across different income levels and investigate the contributions of age, period, and birth cohort effects to this burden. To address these gaps, we utilized data from the Global Burden of Disease, Injury, and Risk Factors Study (GBD) 2021. We analyzed T2DM prevalence data from the GBD 2021 database using the APC and Bayesian APC (BAPC) models to examine trends, assess SDI‐related inequalities, and project future patterns from 2022 to 2030. Through decomposition analysis, we identified key contributing factors and evaluated the impact on women of childbearing age across various SDI levels.

## 2. Methods

### 2.1. Data Sources and Disease Definition

This study utilized GBD 2021 data from the Institute for Health Metrics and Evaluation (IHME), encompassing 371 diseases, 88 risk factors, and data from 204 countries and territories [12, 13]. T2DM, classified as a fourth‐level noncommunicable disease (ICD‐10: E11), is distinguished by subtracting Type 1 diabetes cases from total diabetes prevalence. GBD integrates data from 22,236 sources using advanced methods like MR‐BRT with Bayesian priors and DisMod‐MR 2.1, ensuring standardized, age‐adjusted comparisons. Prevalence estimates, including 95% uncertainty intervals (UIs), were sourced from the Global Health Data Exchange (https://vizhub.healthdata.org/gbd-results/), offering robust measures of variability. Countries are categorized by the SDI into five groups, reflecting development levels and enabling standardized health outcome comparisons [12].

### 2.2. Study Population

The study focused on WCBAs, defined by the WHO as women aged 15–49 who have reproductive capabilities and undergo cyclical hormonal changes. Notably, the study included women aged 15–19, a phase of adolescence, to address the disease burden specific to younger women.

### 2.3. Examination of Temporal Trends in T2DM Prevalence Among WCBAs

The study evaluated the prevalence of T2DM in WCBAs from 1992 to 2021, utilizing case numbers and age‐standardized prevalence rates (ASRs) per 100,000 population, with 95% CIs. Age differences were addressed by age‐standardizing crude rates through the direct method, assuming rates are a weighted sum of independent Poisson random variables.

### 2.4. APC Analysis of T2DM Prevalence in WCBAs

The APC model addresses linear dependency (cohort = period − age) through techniques like maximum likelihood estimation and Bayesian methods, mitigating collinearity for robust parameter estimation. Widely applied in epidemiology, it disentangles biological aging, period‐specific events, and cohort exposures, offering insights into health and socioeconomic trends [4, 14].

Using GBD 2021 data (1992–2021), T2DM prevalence in WCBAs was analyzed across seven age groups (15–49 years), six 5‐year periods, and twelve 10‐year birth cohorts (1942–2021), enabling the separation of age, period, and cohort effects. Log‐linear regression assessed rates, while logistic regression evaluated binary outcomes. Net drift quantified overall trends, and local drift captured age‐specific changes. Age‐specific rates reflected age effects, while period and cohort effects indicated relative prevalence risks, unaffected by reference group selection. In the APC model, the age effect is represented by age‐specific rates for different birth cohorts. The period and cohort effects are represented by the relative risk of prevalence for each period or cohort, which is determined by comparing the age‐specific rates for each to a designated reference period or cohort. It is important to note that the choice of reference period or cohort is arbitrary. While this selection does not affect the interpretation of the results, it is essential to recognize that the comparison is relative to the chosen reference.

### 2.5. Cross‐Country Inequality Analysis

To quantify SDI‐related inequalities in T2DM prevalence among WCBAs, we used the slope of inequality index (SII) for absolute inequality and the concentration index for relative inequality. Positive values indicate a higher burden in high‐SDI countries, while negative values reflect a greater burden in low‐SDI countries. Larger absolute values denote greater disparities [15, 16].

### 2.6. Decomposition and Impairment Analysis

To analyze drivers of T2DM prevalence changes (1992–2021), decomposition analyses examined population growth, aging, and epidemiologic changes [17]. From GBD 2021′s impairment hierarchy, T2DM‐attributable blindness and vision loss data were extracted, categorized as first‐ and second‐level impairments, and modeled to estimate prevalence proportions among WCBAs across SDI levels.

### 2.7. Bayesian Projections

The BAPC model, an enhancement of the traditional APC method, was used to project T2DM prevalence trends (2022–2030). By incorporating Bayesian inference, it offers robust, probabilistic parameter estimation, with nested Laplace approximations ensuring computational efficiency and precision [11].

### 2.8. Ethical Considerations

This study relied solely on publicly available data, which did not necessitate ethical approval, as no personal or confidential information was involved. All methods adhered to the GATHER guidelines, ensuring consistency and transparency [18].

### 2.9. Statistics

Results were expressed as ASRs per 100,000 population, with 95% UIs. A two‐tailed *p* value of less than 0.05 was used to establish statistical significance. All analyses were conducted in R Studio (Version 4.2.1), with specialized R packages, including BAPC, INLA, and ggplot2, used for implementing the APC and BAPC models. The Wald *χ* ^2^ test was used to assess the significance of annual percentage change trends from the APC model [13, 19].

## 3. Results

### 3.1. Trends in T2DM Prevalence in WCBAs, 1992–2021

**Figure 1:**
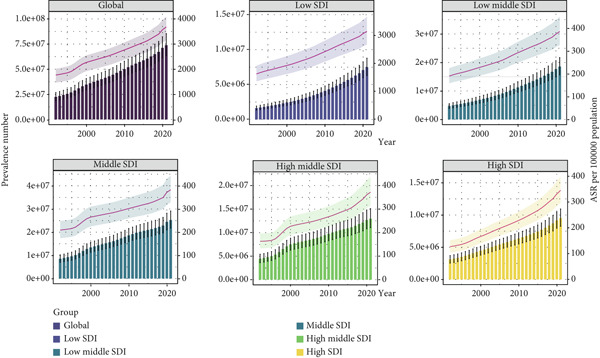
The prevalence numbers and ASR of T2DM in global and the five SDI regions among WCBAs from 1992 to 2021. ASR, age‐standardized prevalence rate; T2DM, Type 2 diabetes mellitus; SDI, sociodemographic index; WCBAs, women of childbearing age.

Global and regional prevalence numbers, ASRs, and net drift in the prevalence of T2DM among WCBAs from 1992 to 2021 are demonstrated in Figure 1 and Table 1. Over this period, the global and regional prevalence of T2DM in this population increased by approximately 2.28%, reaching 73.86 million (95% UI: 63.43–85.32) in 2021. The percentage change in T2DM prevalence increased across all SDI regions. In 2021, the global ASR of T2DM among WCBAs was 3678.6 per 100,000 population (95% UI: 3154.7–4254.5), representing a 1.08% increase since 1992. The ASR increased across all SDI regions: high, high–middle, middle, low–middle, and low. The APC model estimated a global net drift in T2DM prevalence among WCBAs at 2.53% annually (95% CI: 2.49%–2.57%), with regional variations from 2.01% annually (95% CI: 1.94%–2.08%) in middle‐SDI regions to 3.78% annually (95% CI: 3.69%–3.86%) in high‐SDI regions.

**Table 1:** Trends of T2DM prevalence in WCBAs from 1992 to 2021 across SDI quintiles.

**Location**	**1992**		**2021**		**1992–2021 APC**
	**Prevalence number (** **N** **, 95% UI)**	**Prevalence ASR (per 100,000, 95% UI)**	**Prevalence number (** **N** **, 95% UI)**	**Prevalence ASR (per 100,000, 95% UI)**	**Net drift (%/year)**
Global	22,496,858 (18,563,996; 26,870,105)	1766.8 (1467.1, 2099.9)	73,858,922 (63,433,476; 85,320,524)	3678.6 (3154.7, 4254.5)	2.53 (2.49, 2.57)
Low SDI	1,624,940 (1,343,116; 1,933,787)	1631.4 (1361.7, 1923.7)	7,485,015 (6,313,045; 8,782,708)	3154 (2679.1, 3677.5)	2.33 (2.3, 2.36)
Low–middle SDI	4,710,595 (3,889,870; 5,596,286)	1906.3 (1589, 2246.1)	18,565,820 (15,668,658; 21,735,513)	3857.2 (3263.7, 4504.8)	2.48 (2.45, 2.5)
Middle SDI	8,551,312 (7,052,551; 10,219,078)	2090.8 (1739.2, 2481.8)	25,244,095 (21,763,766; 29,137,084)	3829.7 (3292, 4430.6)	2.01 (1.94, 2.08)
High–middle SDI	4,479,853 (3,634,433; 5,421,055)	1635.7 (1333.1, 1973.5)	12,964,107 (11,051,836; 15,112,952)	3720.3 (3148.5, 4364.6)	2.89 (2.78, 3)
High SDI	3,107,365 (2,544,551; 3,703,944)	1272.6 (1038.2, 1522)	9,536,739 (8,251,796; 10,933,224)	3457.1 (2971.8, 3984.9)	3.78 (3.69, 3.86)

*Note:* Parentheses for GBD estimates denote 95% uncertainty intervals, and parentheses for net drift denote 95% CIs.

Abbreviations: 95% CI, 95% confidence interval; 95% UI, 95% uncertainty interval; APC, age period cohort; ASR, age‐standardized prevalence rate; SDI, sociodemographic index; T2DM, Type 2 diabetes mellitus; WCBAs, women of childbearing age.

The national prevalence and ASR in 2021, as well as the net drift of prevalence of T2DM among WCBAs from 1992 to 2021, are shown in Figure 2 and Tables S1 and S2. In 2021, among 204 countries and territories, eight countries reported over 1 million prevalent number: Mexico (2,458,313; 95% UI: 2,076,437–2,866,945), China (17,450,317; 95% UI: 14,840,016–20,383,999), the United States (3,020,792; 95% UI: 2,623,444–3,451,048), Bangladesh (2,418,066; 95% UI: 2,052,168–2,851,355), India (14,500,694; 95% UI: 12,036,671–17,150,827), Pakistan (2,525,387; 95% UI: 2,075,252–3,021,205), Indonesia (1,685,287; 95% UI: 1,367,900–2,031,338), and Brazil (1,505,795; 95% UI: 1,215,481–1,826,271). China, India, and the United States reported the most cases. The global average ASR was 4209.69, with 74 countries exceeding this global average. Six countries, including American Samoa, Cook Islands, Marshall Islands, Niue, Palau, and Samoa, had prevalence rates exceeding three times the global average; notably, all are Pacific island nations with varying SDI levels. Between 1992 and 2021, Greenland saw the largest rise in ASR, with an annual net drift in prevalence of 11.32% (95% UI: 8.33%–14.39%). ASRs increased in all countries and territories, with the APC model estimating increasing trends in net drift for 195 of the 204 countries and territories.

Figure 2World map of (a) ASR in 2021 and (b) net drift of prevalence from 1992 to 2021 for T2DM in WCBAs in 204 countries and territories. ASR, age‐standardized prevalence rate; T2DM, Type 2 diabetes mellitus; WCBAs, women of childbearing age.

**Figure:**
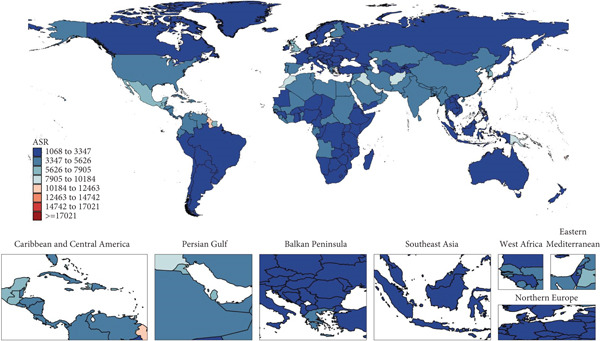
(a)

**Figure:**
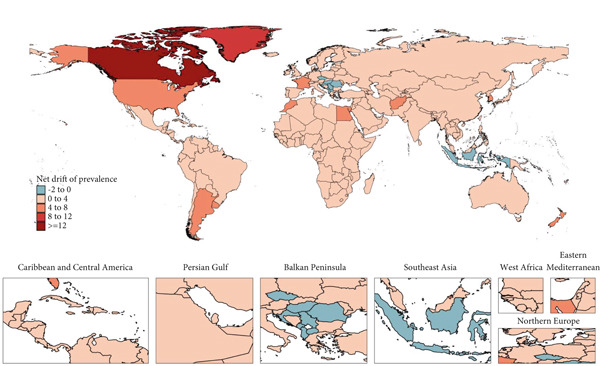
(b)

However, several countries demonstrated a reduction in T2DM burden, with a net drift less than 0: North Macedonia (−0.94%; 95% UI: −3.37% to 1.54%), Montenegro (−1.04%; 95% UI: −3.52% to 1.50%), Czechia (−0.53%; 95% UI: −1.47% to 0.42%), Croatia (−1.7%; 95% UI: −3.19% to −0.18%), Hungary (−0.6%; 95% UI: −1.22% to 0.03%), Romania (−0.37%; 95% UI: −0.93% to 0.2%), Indonesia (−0.68%; 95% UI: −0.89% to −0.47%), and Serbia (−1.81%; 95% UI: −3.71% to 0.13%). Only Albania displayed relatively stable trends, with a net drift of 0% (95% UI: −0.35% to 0.35%). Overall, these findings indicate that the global burden of T2DM among WCBAs is generally on the rise.

### 3.2. Temporal Trends in T2DM Prevalence in WCBAs Across Different Age Groups

**Figure 3:**
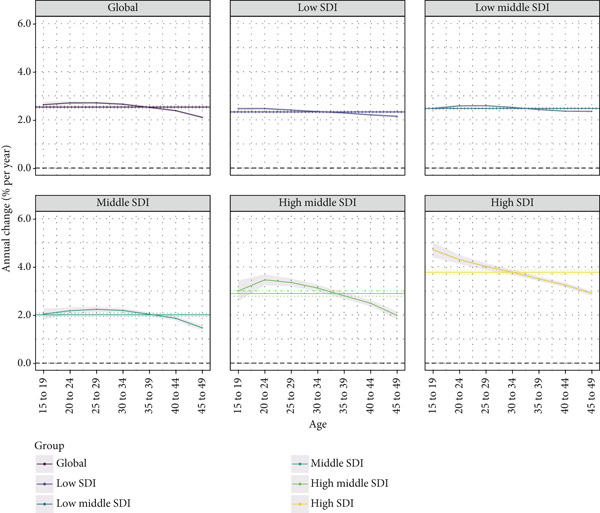
Local drift of prevalence for T2DM in WCBAs across SDI quintiles from 1992 to 2021 for seven age groups. The dots and shaded areas denote the local drift (i.e., annual percentage change of age‐specific prevalence, % per year) and their corresponding 95% CIs. 95% CI, 95% confidence interval; T2DM, Type 2 diabetes mellitus; WCBAs, women of child‐bearing age; SDI, sociodemographic index.

The local drift in the prevalence of T2DM among WCBAs across SDI quintiles from 1992 to 2021 for seven age groups is presented in Figure 3 and Table S3. The prevalence of T2DM among WCBAs has been rising globally across all age groups. The trend increased with age in the 15–19‐year group and stabilized from the 20–24‐year group (2.71%; 95% UI: 2.63%–2.79%) to the 25–29‐year group (2.71%; 95% UI: 2.64%–2.77%). After age 29, the rate of increase gradually declined with age, reaching its lowest level in the 45–49‐year group (2.11%; 95% UI: 2.04%–2.17%).

In all SDI regions, T2DM prevalence among WCBAs rose across age groups, with a declining trend as age increased. Low‐SDI regions showed a steady decline with age, while low–middle‐SDI regions peaked at 25–29 years. Middle‐ and high–middle‐SDI regions peaked at 25–29 and 20–24 years, respectively, before declining. High‐SDI regions showed a consistent age‐related decline. Overall, T2DM prevalence is rising, but the rate slows with age. Table S4 details local prevalence trends.

### 3.3. Analysis of Age, Period, and Birth Cohort Influences on T2DM Prevalence Among WCBAs

Figures S1 and S2 and Tables S5, S6, and S7 illustrate the age, period, and birth cohort effects on T2DM prevalence among WCBAs as determined by the APC model. Age‐related effects showed consistent patterns across SDI regions, with the lowest risk in adolescents (15–19 years) and increasing risk with advancing age. Compared with other regions, the prevalence rate in high‐SDI regions is lower across most age groups.

Period effects indicated a rising prevalence risk across SDI regions, with the low‐SDI region showing generally lower period risks throughout the study period, whereas other regions exhibited more unfavorable period risks over time. The relative period risk in 2017–2021, compared to the 1992–1996 reference period, varied from 1.73 (95% UI: 1.69–1.76) in the middle‐SDI region to 2.58 (95% UI: 2.52–2.64) in the high‐SDI region.

Successive birth cohorts in all SDI regions showed a rising prevalence risk. High‐SDI regions exhibited a marked increase in prevalence across birth cohorts. The relative cohort risk for individuals born between 1997 and 2006, compared to those born in the 1942–1951 reference cohort, varied from 2.24 (95% UI: 2.08–2.41) in the middle‐SDI region to 5.38 (95% UI: 4.89–5.92) in the high‐SDI region. Online Tables S8, S9, and S10 present the age, period, and birth cohort effects on T2DM prevalence among WCBAs for each country.

### 3.4. Health Inequalities in T2DM Burden Among WCBAs Across SDI Levels

Over the past three decades, the prevalence of T2DM among WCBAs has significantly increased globally, accompanied by a substantial rise in health inequality. During this period, the SII increased from 2416.15 (95% UI: 1922.3–2910) in 1992 to 6108.72 (95% UI: 4765.48–7451.96) in 2021, with an annual increase of 15.19% (EAPC: 15.19; 95% UI: 10.41–20.17, Figure S3A). Additionally, the concentration index increased from 0.14 (95% UI: 0.12–0.16) in 1992 to 0.18 (95% UI: 0.16–0.21) in 2021, with an EAPC of 0.14 (95% UI: 0.11–0.18, Figure S3B). These results indicated that the absolute health inequality has worsened, with the T2DM prevalence gap between high‐ and low‐SDI countries widening substantially over the study period. The trends in SII and CI for T2DM in WCBAs from 1992 to 2021 are detailed in Tables S11 and S12.

### 3.5. Key Drivers of the T2DM Health Burden in WCBAs Across Global and SDI Levels

The decomposition of changes in the T2DM prevalence increase in WCBAs globally and across SDI countries is presented in Figure S4 and Table S13. Globally, epidemiological changes are the primary drivers of the increased prevalence of T2DM in WCBAs, followed by population growth, while the contribution of aging is relatively minor. In high‐ and high–middle‐SDI countries, epidemiological changes are the dominant contributors, whereas, in middle‐ and low–middle‐SDI countries, both epidemiological changes and population growth significantly influence the prevalence increase. In low‐SDI countries, population growth is the main driver of the increasing T2DM prevalence among WCBAs.

### 3.6. Global and SDI‐Based Distribution of T2DM‐Related Visual Impairment in WCBAs

The epidemiological distribution of T2DM‐related visual impairment in WCBAs globally and across SDI quintiles in 2021 is shown in Figure S5 and Table S14. The prevalence of moderate vision loss is relatively uniform across all SDI countries, ranging from 5% to 10%, while severe vision loss is consistently low (< 5%). However, the burden of blindness and vision loss exhibited clear disparities. The highest rates were observed in middle‐SDI regions, which were substantially higher than those in other regions.

### 3.7. Projected Trends and Risk Dynamics in T2DM Prevalence Among WCBAs Across SDI Levels (2022–2030)

To capture global T2DM trends among WCBAs, ASR and prevalence projections (2022–2030) are analyzed in Figures S6 and S7. In high‐SDI countries, Canada′s prevalence is rising. Czechia, despite a higher recent prevalence, shows moderate projections and is the only high‐SDI country with a net drift below 0%. In middle‐SDI countries, China and India exhibit consistent prevalence growth. Among low‐SDI countries, Afghanistan shows the highest net drift with widespread risk increases, while Rwanda exhibits minimal net drift, and its prevalence rate is predicted to rise.

## 4. Discussion

Diabetes is the world′s seventh‐largest noncommunicable disease, posing a severe threat to human health [1]. T2DM constitutes about 90% of diabetes cases [2], imposing a considerable societal burden and significant health risks for WCBAs [20]. T2DM patients face elevated risks of microvascular and macrovascular complications [21], nonalcoholic fatty liver disease [22], and increased mortality from Alzheimer′s disease and related dementias, particularly in postmenopausal women [23]. Women aged 15–49 with T2DM face numerous health challenges, including blood sugar control, cardiovascular risk, neuropathy, kidney disease, retinopathy, self‐management difficulties, and reproductive health concerns [24]. A comprehensive understanding of T2DM prevalence trends among WCBAs is essential to assess the impact of T2DM in this population. A comprehensive analysis of T2DM prevalence in WCBAs is currently unavailable. Although GBD 2021 [11] offers a broad overview of global T2DM prevalence, it fails to specifically address the burden of T2DM in WCBAs, a distinct and critically important group. This omission is significant because T2DM in WCBAs has unique implications, including direct effects on reproductive outcomes, maternal health, and intergenerational disease risk. The primary contribution of our research is the focused analysis of T2DM in this demographic, revealing trends that are often obscured in broader reports that combine all age and gender categories. By isolating WCBAs, we are able to provide a clearer understanding of how T2DM specifically impacts this group and the associated long‐term consequences.

The GBD 2019 study reported a global rise in metabolic disease incidence (2000–2019), particularly in high‐SDI countries, with unchanged T2DM mortality [25]. In low–middle‐SDI regions, T2DM showed the largest increases in incidence, prevalence, mortality, and DALYs [26]. GBD 2021 data revealed rising age‐standardized prevalence and percentage change of T2DM in WCBAs across all SDI regions (1992–2021). By 2021, eight countries exceeded 1 million T2DM cases, with China, India, and the United States ranking highest. Despite advanced healthcare, the United States exhibited disproportionately high prevalence. Dietary risks (e.g., low fruit intake and high red/processed meat consumption) contributed to 26.07% of T2DM mortality and 27.08% of DALYs in 2019, correlating positively with SDI levels [27]. The global average T2DM prevalence in WCBAs in 2021 was 4209.69 per 100,000, with 74 countries exceeding this level. The high prevalence of T2DM in six Pacific island countries—more than three times the global average—cannot be fully explained by the SDI model alone. This elevated prevalence is likely influenced by strong genetic factors, particularly the “thrifty genotype” hypothesis, which suggests that populations developed the ability to store energy more efficiently in response to historical cycles of feast and famine. While this adaptation was advantageous during times of food scarcity, it may now contribute to the heightened risk of T2DM in environments with abundant, calorie‐dense foods [28, 29]. Additionally, the rapid dietary transition in these regions, marked by a shift toward energy‐dense and processed foods [30], has further exacerbated the risk of T2DM. These findings underscore the importance of considering local genetic and sociocultural factors, which may either replace or amplify global trends, offering a more nuanced understanding of the factors driving the high T2DM prevalence in these populations.

Using the APC model, our study highlights the influence of age, period, and cohort effects on T2DM prevalence in WCBAs. Age‐related patterns show that adolescents (15–19 years) have the lowest risk of T2DM, with prevalence rising significantly as individuals age, even among those with normal BMI [31]. However, the upward trend in period and cohort effects reveals that even the youngest group (15–19 years old) is experiencing a concerning increase in T2DM risk. This early onset is particularly alarming, as it signifies prolonged exposure to hyperglycemia, which can have long‐term effects on fertility and overall health [32]. The period effects show an increasing risk of T2DM across SDI regions, with lower risks observed in low‐SDI regions compared to high‐SDI regions. Cohort effects reveal rising T2DM prevalence in successive birth cohorts, with the greatest increase in high‐SDI regions. For example, relative cohort risk for those born between 1997 and 2006 compared to those born in 1942–1951 ranged from 2.24 (95% UI: 2.08–2.41) in middle‐SDI regions to 5.38 (95% UI: 4.89–5.92) in high‐SDI regions. While our data did not directly assess causality, the significantly higher period and cohort risks in high‐SDI areas are closely linked to the obesity epidemic, likely driven by lifestyle and dietary factors. Urbanization, with its associated increase in sedentary behavior and reduced physical activity, along with the growing consumption of ultraprocessed foods, has contributed to higher obesity rates [33, 34], which in turn increases T2DM risk. Furthermore, enhanced screening and diagnostic intensity in developed healthcare systems may lead to the detection of previously undiagnosed cases, inflating the disease incidence [35]. In contrast, the rising T2DM burden in low‐SDI regions is primarily driven by population growth, highlighting challenges related to healthcare system capacity and accessibility. These regions face significant barriers in managing the growing number of cases, which require a strengthened healthcare infrastructure. The decomposition of driving factors based on SDI levels provides a more nuanced understanding of the global T2DM burden than individual overall prevalence data. This analysis suggests that effective policy responses must be tailored to specific contexts: focusing on prevention and lifestyle interventions in high‐SDI environments while strengthening basic medical infrastructure in low‐SDI regions.

This study analyzed future T2DM trends among WCBAs across SDI levels using representative countries. In high‐SDI countries, Canada’s prevalence projects significant future growth. Conversely, the Czech Republic shows moderate projected incidence. Middle‐SDI countries like China and India exhibit consistent growth, indicating a substantial future disease burden. In low‐SDI countries, Afghanistan shows the highest net drift, while Rwanda shows the lowest; however, T2DM risk among WCBAs is projected to gradually increase in both nations. Eight countries (North Macedonia, Montenegro, the Czech Republic, Croatia, Hungary, Romania, Serbia, and Indonesia) showed a decreasing trend in T2DM prevalence among WCBAs, indicating a negative net drift. For the European countries, this trend is likely linked to the success of national public health campaigns targeting obesity reduction and healthier lifestyles [36–39]. In Indonesia, a country with a low‐to‐medium SDI, the decline may be attributed to dietary patterns rich in plant‐based foods, which can help reduce T2DM risk [40]. However, it is important to note that data artifacts cannot be entirely ruled out. These positive trends highlight that the concerning period and cohort effects observed elsewhere are not inevitable. With targeted policy interventions—such as food and environmental regulations, as well as public health education—these negative trends can be reversed.

This study highlights a significant global increase in the prevalence of T2DM among WCBAs during 1992–2021, accompanied by a substantial rise in health inequality. The SII more than doubled from 1992 to 2021, with an annual increase of 15.19%, reflecting a widening absolute disparity, particularly concentrated in high‐SDI countries. Similarly, the CI increased, indicating both a growing disease burden and increasing inequity in distribution. The disproportionate burden in high‐SDI countries may be driven by higher rates of obesity, unhealthy lifestyles, and improved diagnostics [41, 42]. This highlights the need for high‐SDI countries to increase focus on T2DM among WCBAs by adjusting health policies and promoting healthier lifestyles. Our findings lead to several actionable insights. First, the alarming rise in T2DM risk among the youngest WCBAs (15–19 years old) underscores the need for improved primary prevention. This highlights the importance of targeted school‐based and adolescent health promotion programs. To align with SDG 3.4, which aims to reduce premature mortality from noncommunicable diseases, we recommend strengthening T2DM screening within routine reproductive health care, implementing prepregnancy counseling, and integrating maternal care with chronic disease management. Furthermore, our research provides an epidemiological foundation for bundling diabetes care with sexual and reproductive health services. This integrated approach not only addresses T2DM but also improves overall reproductive health, offering a dual return on investment and creating a more holistic framework for public health intervention.

Disease burden drivers varied by SDI level: Epidemiological changes dominated in high‐ and high–middle‐SDI countries, while population growth also contributed significantly in middle‐ and low–middle‐SDI regions. In low‐SDI countries, rapid population growth was the primary factor, exacerbating health inequalities due to limited resources. T2DM‐related visual impairment was disproportionately higher in middle‐SDI regions, with severe vision loss and blindness emphasizing the need for early screening and intervention. Despite global improvements in relative health inequality for T2DM, absolute inequality is worsening, particularly in low‐SDI regions, underscoring the urgency of enhancing resource allocation and prevention strategies to reduce disparities.

Our study makes several key advancements in understanding the global burden of T2DM in WCBAs (15–49 years old), extending beyond the general findings of the GBD reports [11]. First, by focusing exclusively on WCBAs, we highlight the unique and growing burden of T2DM in this vulnerable group, which presents specific risks to reproductive health and carries intergenerational consequences. This targeted focus allows us to capture the specific health challenges faced by women in this age group, offering a clearer picture of the disease′s impact. Second, the application of the APC model helps us disentangle the independent effects of age, period, and birth cohort, providing deeper insights into the factors driving the rising incidence of T2DM. By examining these effects, we can better understand the long‐term trends shaping the disease burden, particularly in the context of WCBAs. Third, by incorporating socioeconomic inequality indicators and Bayesian forecasting, we not only quantify the widening gap in T2DM prevalence across socioeconomic strata but also predict the future trajectory of these disparities. This approach offers valuable insights into the sustainability of the socioeconomic gap and its potential implications for public health. Finally, the decomposition analysis allows us to pinpoint the key factors driving differences in T2DM burden across various SDI environments. This evidence‐based understanding provides a strong foundation for developing tailored policy interventions aimed at addressing the specific drivers of T2DM in different regions.

This study is the first to analyze T2DM prevalence trends among WCBAs across 204 countries using GBD 2021 data and the APC model. Building on prior GBD 2019 analyses of metabolic diseases and T2DM mortality trends [43], it provides a comprehensive examination of prevalence patterns across SDI regions and age groups. The APC model enabled detailed assessment of temporal trends by period and birth cohort, offering valuable insights for global T2DM epidemiology and health policy. This study has inherent limitations due to the GBD framework. In addition to the time lag in data, a more critical issue is the modeling approach, which relies on diverse data sources of varying quality. In particular, the scarcity of raw data in many low‐income countries results in estimates that are uncertain and heavily dependent on covariate forecasting and regional imputation. These methods may obscure local realities, meaning that findings for these regions reflect modeled estimates rather than empirical data and should therefore be interpreted with caution. Another limitation is the use of national‐level data, which can lead to ecological fallacies. Associations observed at the group level may not accurately reflect individual risks, and this discrepancy should be considered when interpreting the results. Additionally, because we did not apply the spatially correlated Poisson model in our APC analysis, overdispersion and correlations between age, period, and cohort effects must also be taken into account when interpreting the findings [44]. Regarding the Bayesian projections to 2030, validating these projections with external datasets is essential to strengthen their robustness. Further validation against out‐of‐sample data would provide greater confidence in their accuracy.

In summary, this study indicates that the prevalence of T2DM among WCBAs is increasing globally, with prevalence rates expected to continue rising in many countries and regions, and the disease burden will become increasingly concentrated in high‐SDI countries. The health of WCBAs is closely tied to population growth and social development, highlighting an urgent need for increased investment in T2DM healthcare resources, expanded research on treatment strategies, and strengthened health policies.

## Consent

The authors have nothing to report.

## Disclosure

All authors have read and approved the final version of the manuscript. A preprint has previously been published [45]. Patients and/or the public were not involved in this research.

## Conflicts of Interest

The authors declare no conflicts of interest.

## Author Contributions

M.C., Z.X., M.L., J.Q., and F.W. designed the study and drafted the manuscript. M.L., X.S., Q.Y., and X.H. performed the statistical analysis. Y.Z., Y.C., and F.W. assisted with the interpretation of the results and funding. All authors contributed to the acquisition, analysis, or interpretation of data. All authors discussed the results, revised the report, and approved the final version before submission. Z.X. and M.L. contributed equally to this work.

## Funding

This study was funded by the Key Research and Development Program of Hebei Health Co, 20231340, and the Medical Science Research Project of Hebei Health Commission, 20240368.

## Supporting Information

Additional supporting information can be found online in the Supporting Information section.

## Supporting information


**Supporting Information 1** Figure S1: Age, period, and birth cohort effects on T2DM prevalence in WCBAs by APC models across SDI quintiles. Figure S2: Local drift, age, period, and birth cohort effects on T2DM prevalence in WCBAs by APC models in exemplary countries. Figure S3: Inequality in prevalence of T2DM among WCBAs, 1992–2021. Figure S4: Decomposition analysis results for the global population and five SDI regions. Figure S5: Proportion of prevalent cases of impairments attributable to T2DM among WCBAs by global and SDI levels in 2021. Figure S6: Projects the ASR and prevalence numbers for T2DM in WCBAs globally from 2022 to 2030. Figure S7: Projects the ASR and prevalence numbers for T2DM in WCBAs in exemplary countries from 2022 to 2030.

**Supporting Information 2** Table S1: T2DM prevalence numbers and ASR in WCBAs from 1992 to 2021 in 204 countries and territories. Table S2: APC analysis of T2DM prevalence in global and five SDI regions among WCBAs from 1992 to 2021. Table S3: The local drift of prevalence from 1992 to 2021 for T2DM in WCBAs across SDI quintiles for seven age groups. Table S4: The local drift of prevalence from 1992 to 2021 for T2DM in WCBAs for seven age groups across countries. Table S5: Age effects on T2DM prevalence in WCBAs across SDI quintiles. Table S6: Period effects on T2DM prevalence in WCBAs across SDI quintiles. Table S7: Cohort effects on T2DM prevalence in WCBAs across SDI quintiles. Table S8: Age effects on T2DM prevalence in WCBAs across countries and territories. Table S9: Period effects on T2DM prevalence in WCBAs across countries and territories. Table S10: Cohort effects on T2DM prevalence in WCBAs across countries and territories. Table S11: Trends in absolute inequality index (SII) and its estimated annual percentage change (EAPC) across global levels. Table S12: Trends in concentration index (CI) and its estimated annual percentage change (EAPC) across global levels. Table S13: Decomposition of overall health burden differences by aging, population growth, and epidemiological changes across SDI and global levels. Table S14: Proportion of prevalent cases of impairments attributable to T2DM among WCBAs by global and SDI levels in 2021.

## Data Availability

These data were derived from the following resources available in the public domain: the Global Health Data Exchange, https://vizhub.healthdata.org/gbd-results.
