# Supplementary material for: Global, Regional, and National Prevalence for Type 2 Diabetes Among Women of Childbearing Age, 1992–2021: An Age–Period–Cohort Analysis Based on the Global Burden of Disease Study 2021
Source: J Diabetes Res. 2026 Jan 29;2026:2197672. doi: 10.1155/jdr/2197672 (PMC12853072; doi:10.1155/jdr/2197672)
Supplement: Supplementary file 1 — Supporting Information 1 Figure S1: Age, period, and birth cohort effects on T2DM prevalence in WCBAs by APC models across SDI quintiles. Figure S2: Local drift, age, period, and birth cohort effects on T2DM prevalence in WCBAs by APC models in exemplary countries. Figure S3: Inequality in prevalence of T2DM among WCBAs, 1992–2021. Figure S4: Decomposition analysis results for the global population and five SDI regions. Figure S5: Proportion of prevalent cases of impairments attributable to T2DM among WCBAs by global and SDI levels in 2021. Figure S6: Projects the ASR and prevalence numbers for T2DM in WCBAs globally from 2022 to 2030. Figure S7: Projects the ASR and prevalence numbers for T2DM in WCBAs in exemplary countries from 2022 to 2030. [file JDR-2026-2197672-s002.docx]

**Supplementary Materials**

**Supplementary Figure1 Age, period and birth cohort effects on T2DM prevalence in WCBA by APC models across SDI quintiles.**
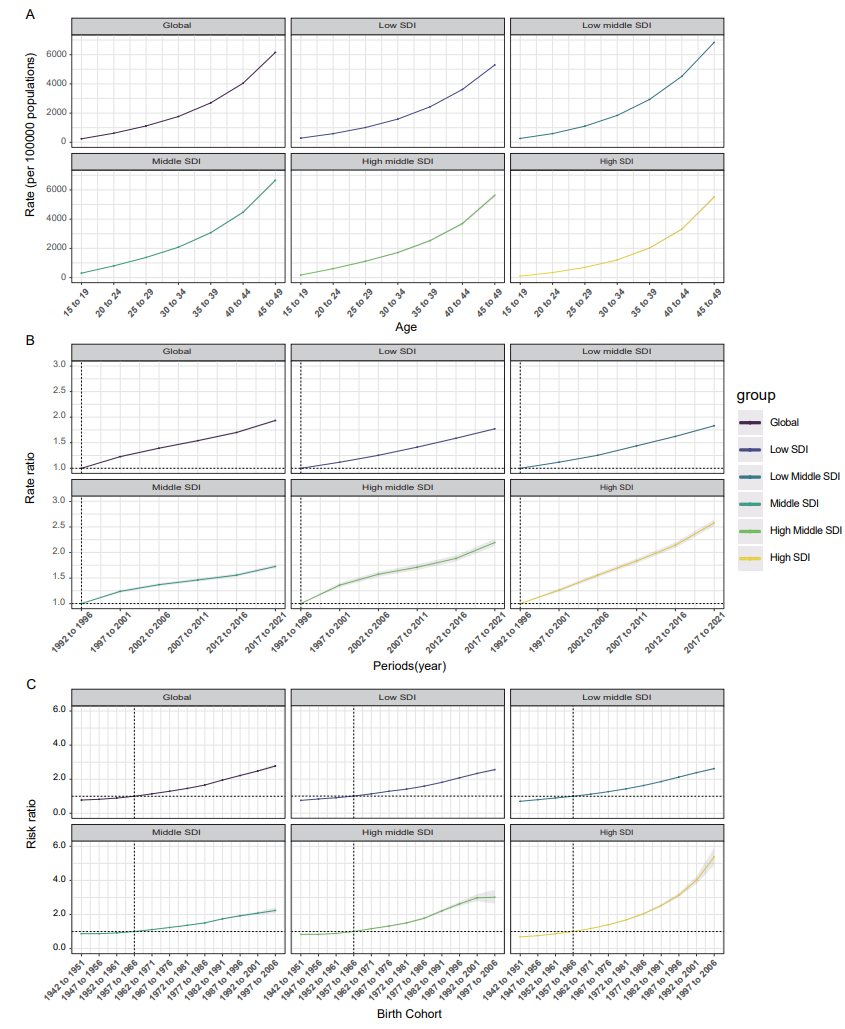


1. The age effect are illustrated by the longitudinal rates specific to age, which are adjusted for variations across different birth cohorts, taking into account the period-specific deviations. (B) Period effects are shown through the relative risk of T2DM prevalence during different periods, calculated as the ratio of the age-specific rates from the period from 1992 - 1996 to 2017 - 2021, with the baseline period set as 1992 - 1996. (C) Birth cohort effects are demonstrated by the cohort relative risk of prevalence and calculated as the ratio of age-specific rates from 1942–1951 cohort to 1997–2006 cohort, with the reference cohort set at 1972–1981. The dots and shaded areas denote the prevalence rates or rate ratios and their corresponding 95% CIs. 95% CI, 95% Confidence Interval; T2DM, type 2 diabetes mellitus; WCBA, women of child-bearing age; APC, age period cohort; SDI, sociodemographic index.

**Supplementary Figure2 Local drift, age, period and birth cohort effects on T2DM prevalence in WCBA by APC models in exemplary countries.**
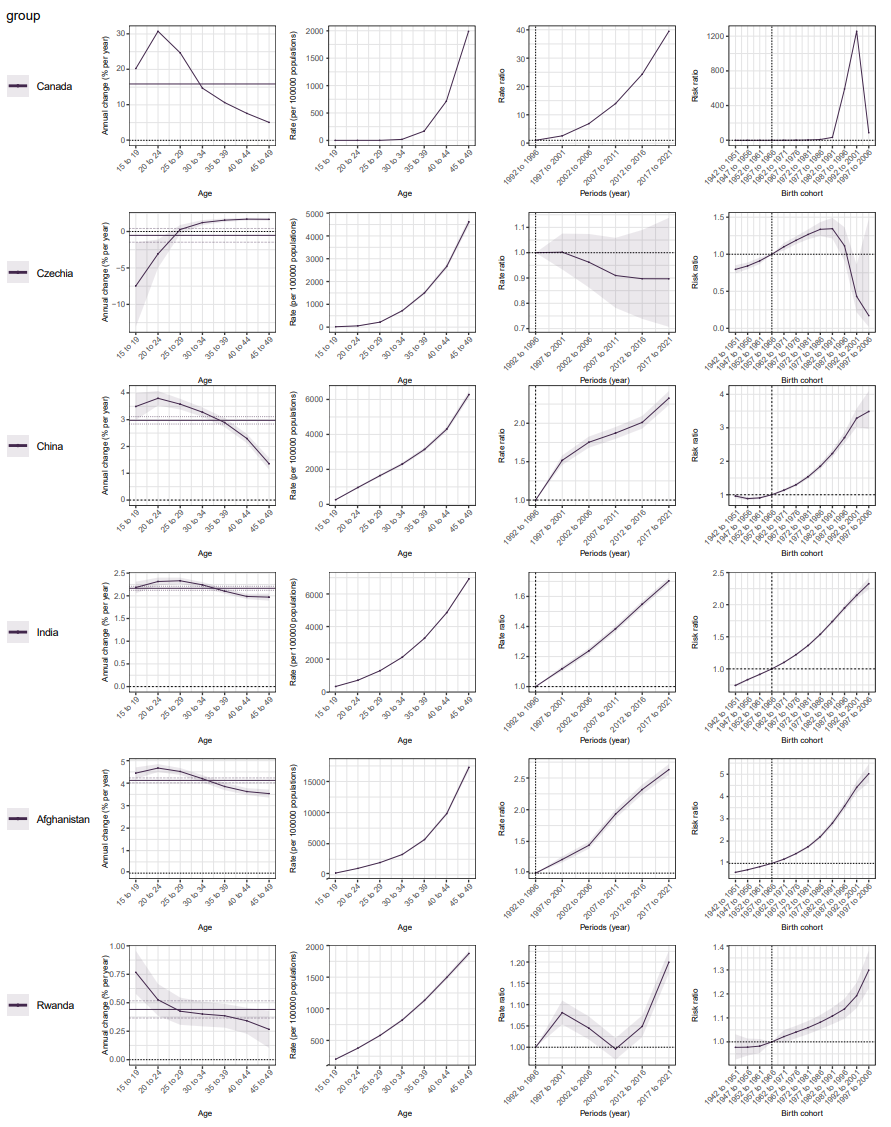


Local drift denotes the annual percentage change of age-speciﬁc prevalence (% per year) from 1990 to 2019 for seven age groups (15–19, 20–24, 25–29, 30–34, 35–39, 40–44, 45–49 years). The age effect are illustrated by the longitudinal rates specific to age, which are adjusted for variations across different birth cohorts, taking into account the period-specific deviations. Period effects are shown through the relative risk of T2DM incidence during different periods, calculated as the ratio of the age-specific rates from the period from 1992 - 1996 to 2017 - 2021, with the baseline period set as 1992 - 1996. Birth cohort effects are demonstrated by the cohort relative risk of incidence and calculated as the ratio of age-specific rates from 1942–1951 cohort to 1997–2006 cohort, with the reference cohort set at 1972–1981. The dots and shaded areas denote the incidence rates or rate ratios and their corresponding 95% CIs. 95% CI, 95% Confidence Interval; T2DM, type 2 diabetes mellitus; WCBA, women of child-bearing age; APC, age period cohort; SDI, sociodemographic index.

**Supplementary Figure3 Inequality in prevalence of T2DM among WCBA, 1992–2021.**
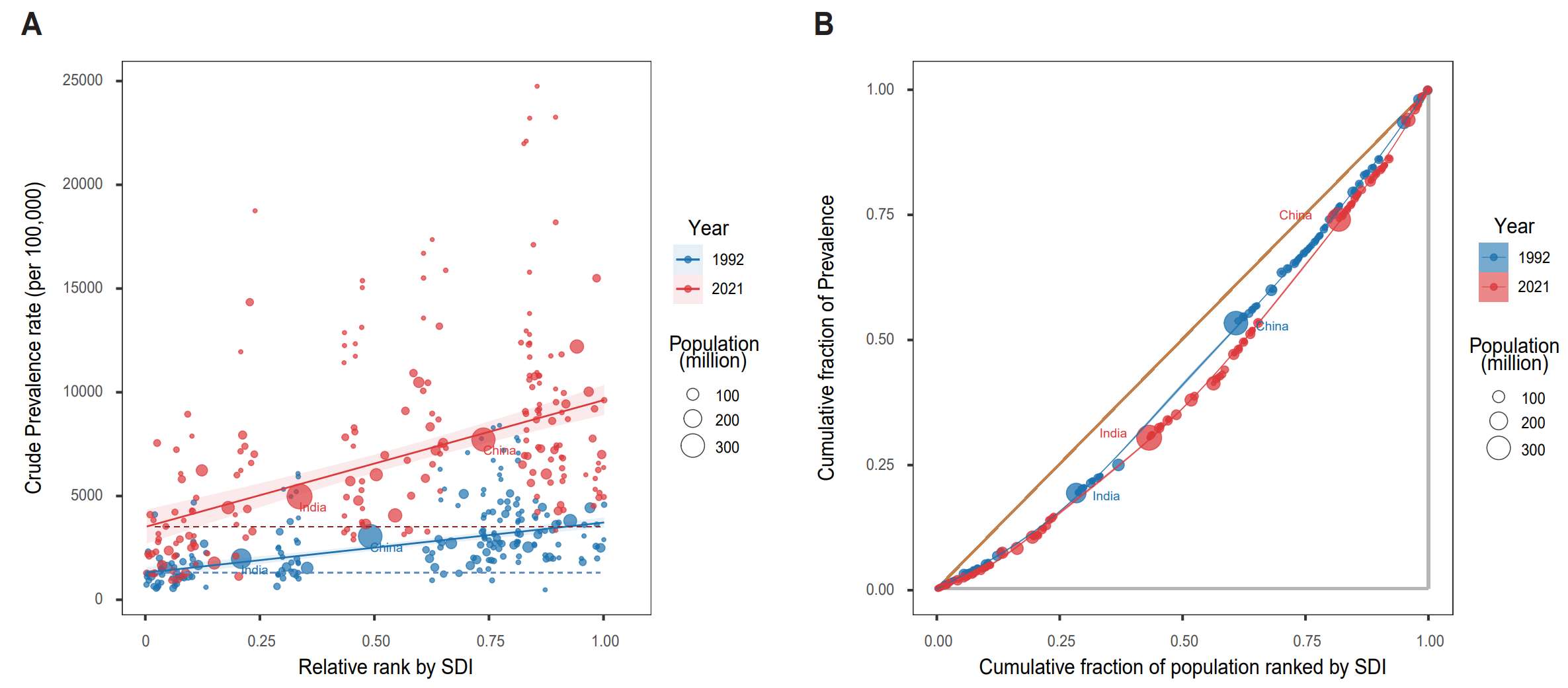


(A) Health inequality regression curves for prevalence of T2DM among WCBA. (B) Concentration curves for prevalence of T2DM among WCBA. Δ, the percentage change of inequality from 1992 to 2021. T2DM, type 2 diabetes mellitus; WCBA, women of childbearing age. SDI, socio-demographic index. SII, slope index of inequality. CI, concentration index. GBD, global burden of disease.

**Supplementary Figure4 Decomposition analysis results for the global population and five SDI regions.**
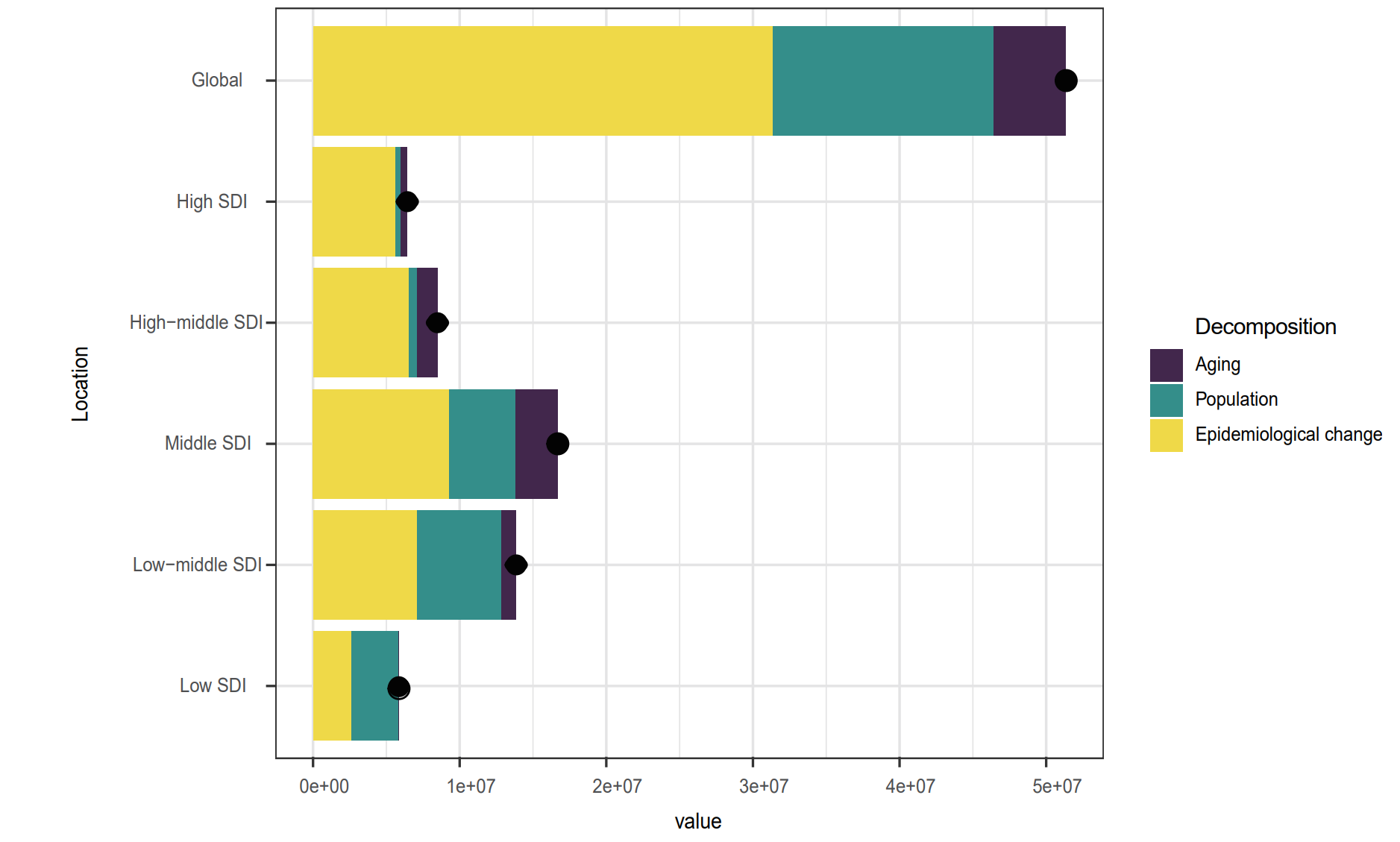


SDI, socio-demographic index.

**Supplementary Figure5 Proportion of prevalent cases of impairments attributable to T2DM among WCBA by global and SDI levels in 2021.**


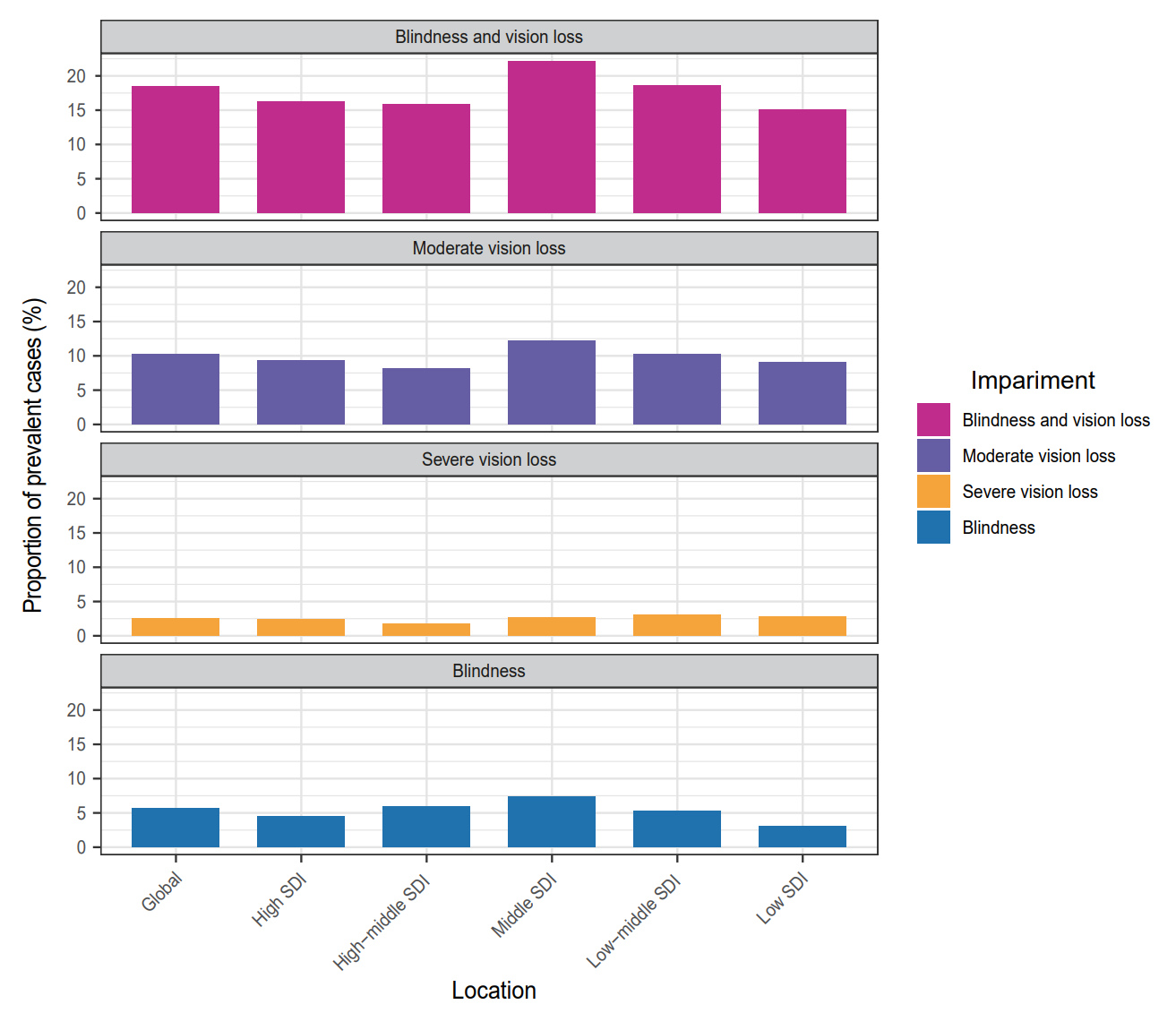


T2DM, type 2 diabetes mellitus; WCBA, women of childbearing age. SDI, socio-demographic index.

**Supplementary Figure6 Projects the ASR and numbers of prevalence for T2DM in WCBA globally from 2022 to 2030.**


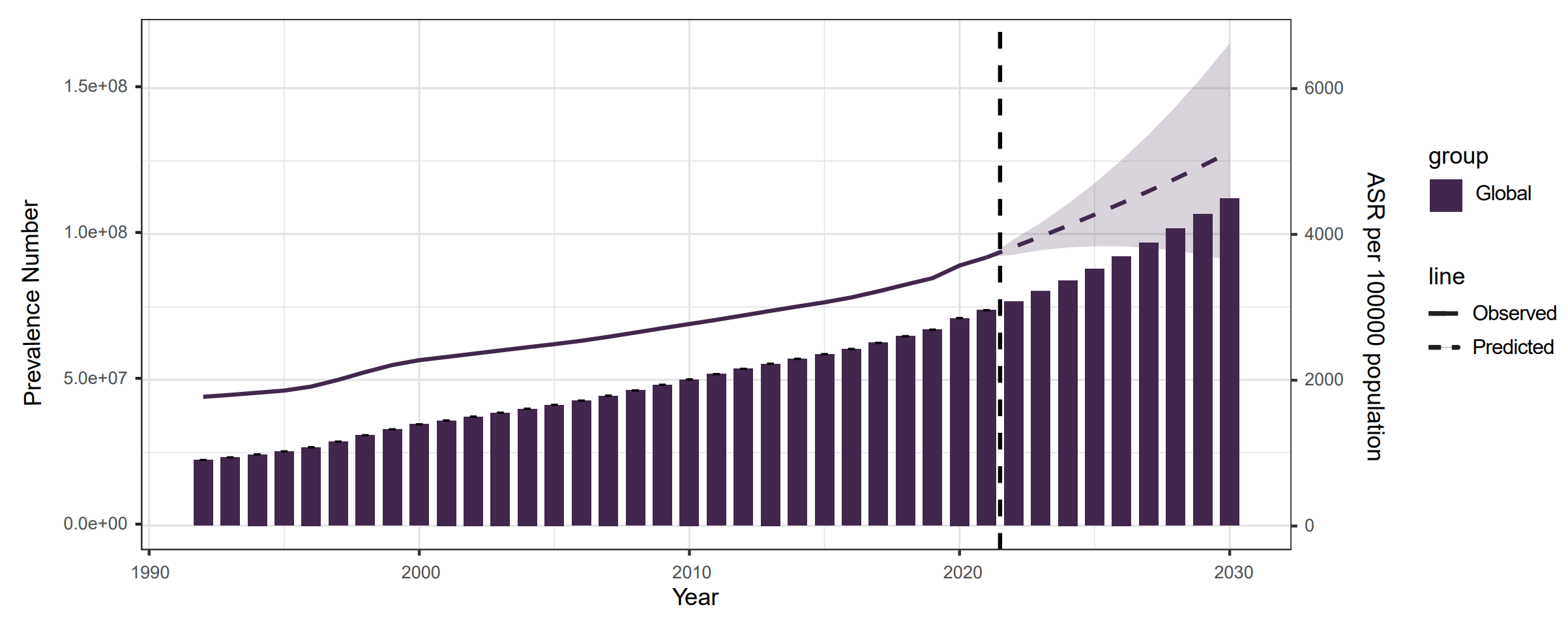


ASR, age-standardized prevalence rate; T2DM, type 2 diabetes mellitus; WCBA, women of childbearing age.

**Supplementary Figure7 Projects the ASR and numbers of prevalence for T2DM in WCBA in exemplary countries from 2022 to 2030.**


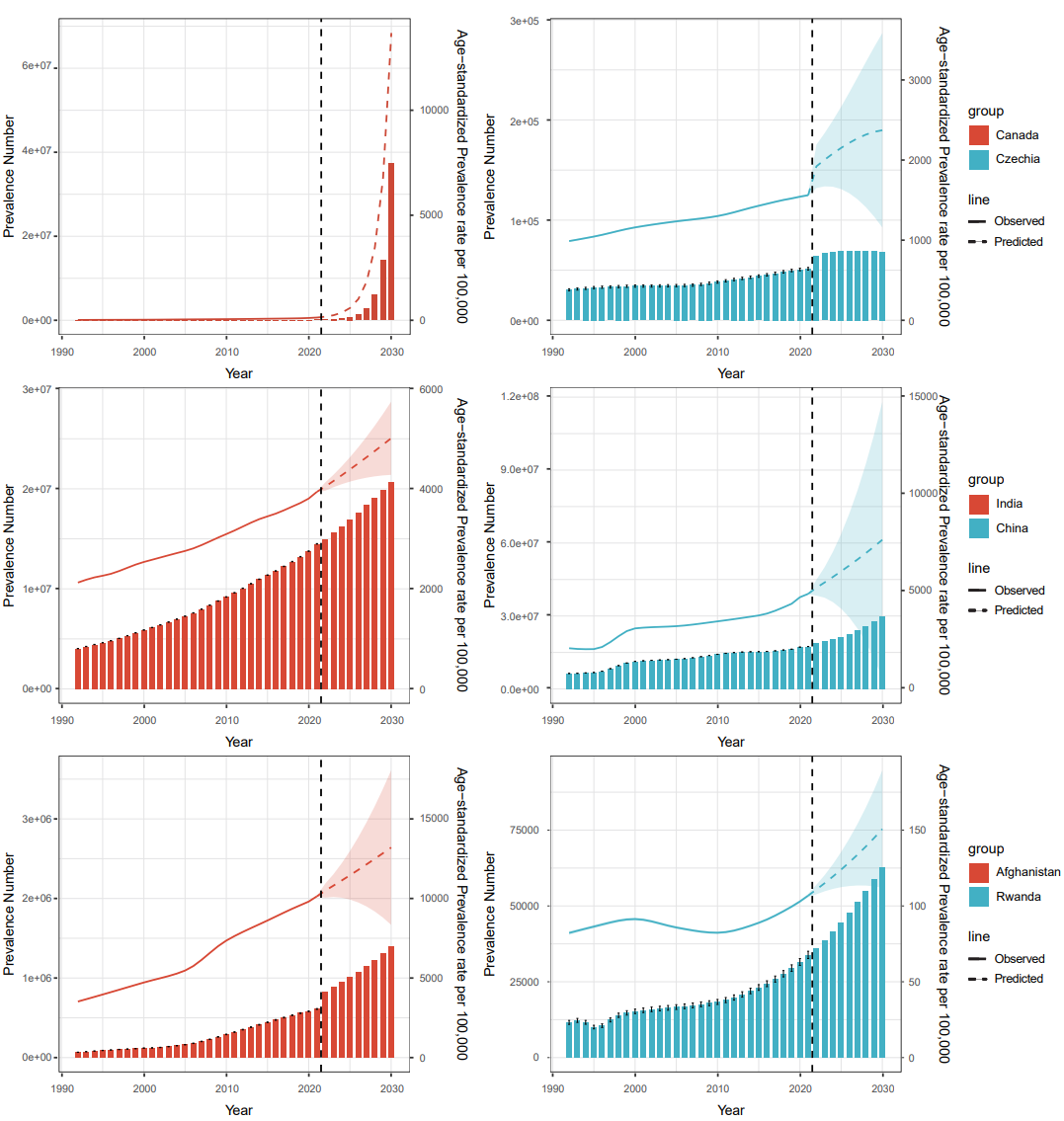


ASR, age-standardized prevalence rate; T2DM, type 2 diabetes mellitus; WCBA, women of childbearing age.
